# Comparative Analysis of Anomaly Detection Approaches in Firewall Logs: Integrating Light-Weight Synthesis of Security Logs and Artificially Generated Attack Detection †

**DOI:** 10.3390/s24082636

**Published:** 2024-04-20

**Authors:** Adrian Komadina, Ivan Kovačević, Bruno Štengl, Stjepan Groš

**Affiliations:** Faculty of Electrical Engineering and Computing, University of Zagreb, 10000 Zagreb, Croatia; ivan.kovacevic@cyberarrange.com (I.K.); bruno.stengl@fer.hr (B.Š.); stjepan.gros@fer.hr (S.G.)

**Keywords:** cybersecurity, datasets, security logs, firewall logs, artificially generated attacks, machine learning, anomaly detection

## Abstract

Detecting anomalies in large networks is a major challenge. Nowadays, many studies rely on machine learning techniques to solve this problem. However, much of this research depends on synthetic or limited datasets and tends to use specialized machine learning methods to achieve good detection results. This study focuses on analyzing firewall logs from a large industrial control network and presents a novel method for generating anomalies that simulate real attacker actions within the network without the need for a dedicated testbed or installed security controls. To demonstrate that the proposed method is feasible and that the constructed logs behave as one would expect real-world logs to behave, different supervised and unsupervised learning models were compared using different feature subsets, feature construction methods, scaling methods, and aggregation levels. The experimental results show that unsupervised learning methods have difficulty in detecting the injected anomalies, suggesting that they can be seamlessly integrated into existing firewall logs. Conversely, the use of supervised learning methods showed significantly better performance compared to unsupervised approaches and a better suitability for use in real systems.

## 1. Introduction

This paper is an extension of two papers originally presented at the 17th International Conference on Telecommunications (ConTEL) © 2023 IEEE [1] and the 16th European Workshop on System Security [2]. Parts of this paper appeared in Proceedings of the 17th International Conference on Telecommunications (ConTEL) © 2023 IEEE [1] and Proceedings of the 16th European Workshop on System Security [2].

In many large networks, detecting anomalies in traffic patterns is an important task. However, the definition of what counts as an anomaly remains difficult, with different researchers proposing their own interpretations [3,4,5,6]. An anomaly is usually defined as points in certain time steps where the system’s behaviour is significantly different from the previous normal status [7]. These anomalies in the network can come from a variety of sources, from expected deviations in network traffic manifesting as statistical outliers within normal network behavior to deliberate actions taken by malicious actors operating on the network. Particularly sophisticated attacks attempt to conceal their actions and presence. To avoid detection, attackers attempt to disguise themselves and evade easy identification through simple network traffic statistics or attribute value distributions.

To overcome this challenge, anomaly detection systems have proven to be an important solution [8]. These systems are designed to detect whether the test data match the expected distributions of normal data and flag anomalies as non-conforming points [7]. They are capable of detecting both known and previously unknown attacks or malicious behaviors.

Logs serve as one of the most important sources of information in the search for anomalies. In today’s technology landscape, systems routinely generate various logs resulting from their use. Firewall logs in particular contain important insights into the network structure of the network and the typical flow of network traffic. Thorough analysis of these logs is essential to extract as much relevant network-related information as possible, especially in large networks where many traffic patterns remain unknown.

When developing intrusion detection methods, the validation and evaluation of log analysis methods is of utmost importance. These methods aim to detect signs of cyberattacks, distinguish benign events from false positives, and categorize events and alerts based on their underlying causes. An overview of strategies to achieve the latter goal can be found in the comprehensive work of Kovačević et al. [9].

**Figure 1:**
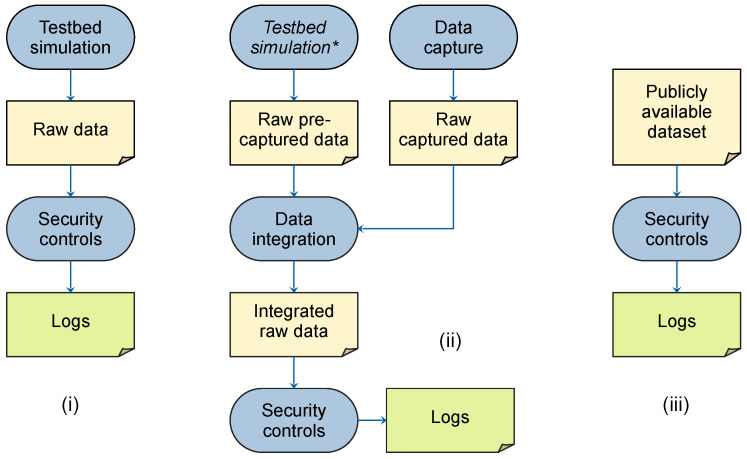
Methods of log synthesis used in previous research. Rounded rectangles represent processes whose inputs and outputs are represented by document symbols and connected by arrows. Method (**i**) uses a testbed, method (**ii**) integrates pre-captured artifacts, which could have been created using a testbed, into a set of captured network traffic, and method (**iii**) loads captured network traffic from an available dataset. All methods input raw data into security controls to obtain logs. * The step of creating artifacts in the testbed is optional if they are obtained by other methods.

After an extensive review of the existing literature, it was found that most methods for analyzing logs and correlating alerts rely heavily on very detailed datasets for validation purposes [2]. These datasets typically consist of raw network traffic or event data, often referred to as low-level data. Essentially, the above approaches use one of three methods to generate logs, shown in Figure 1:

1. Use of a controlled testbed equipped with security controls to generate logs [10,11].
2. Integrating pre-captured artifacts from a testbed environment with recorded network traffic [12,13] and inputting this combined dataset into a security control to generate logs.
3. Inputting previously recorded network traffic from a dataset into a security control to generate logs [14]. It can be noted that similar methods have also seen frequent use in the evaluation of various alert correlation approaches. For instance, Ning et al. [15] used the RealSecure Network Sensor to generate IDS alert logs based on an existing public dataset.

All three methods have considerable limitations. The first method requires setting up a special testbed capable of generating network traffic. As shown in the work of Sharafaldin et al. [10], such testbeds must accurately simulate user interaction, making this method difficult to implement in practice [11]. Not only is this approach costly, but it also increases the likelihood of artifacts being introduced and does not necessarily provide a dataset that is realistic or relevant to the target organization. It also raises the question of how such an approach can be generalized based on a constructed testbed. Another important issue is that network traffic patterns change over time [10], so testbeds need to be updated frequently to maintain accuracy.

The second method involves integrating attack artifacts into recorded events or network traffic. These artifacts are usually captured with a dedicated testbed or loaded from a previous recording. The integration of these two datasets requires considerable manual effort, as both datasets contain numerous technical details. Neglecting these details could lead to unintended anomalies, such as protocol-specific counters or unexpected IP addresses or ports for the target network. Salazar et al. [12] used such an approach by developing *5greplay*, a system that allows pre-recorded traffic to be integrated with actual 5G network traffic according to user-defined rules. The combined traffic can then be used for various tasks, such as security testing of a system.

The third method inherits most of the disadvantages of the first two methods. In addition, publicly available datasets have their own drawbacks. These drawbacks include the presence of outdated attacks and network traffic patterns [11], as well as unrepresentative statistical properties [14].

This paper presents a novel method for creating logs containing attack-related records. This approach utilizes real-world logs from the organization and domain knowledge and eliminates the need for a dedicated testbed or installed security controls. A key advantage of this method is that it should produce logs that are very similar to those that would be generated in the original environment, making it more representative compared to the previously mentioned methods. To demonstrate the application of the proposed method, we have used real-world internal firewall logs from a national electricity transmission system operator. Since these logs did not contain any anomalies in their original form, we applied the proposed method to create attack-related records and integrate them with the pre-existing logs. These attacks were primarily network scans tailored to the target environment and involved multiple attempts to establish connections that would be detectable in the firewall logs. Various anomaly detection methods are then applied to the generated logs, including both unsupervised and supervised approaches.

Detecting anomalies based solely on firewall logs raises several questions. First, how to effectively represent firewall log data, i.e., how to construct features from available attributes. Second, whether to use supervised or unsupervised machine learning techniques and which specific algorithm to select from the wide range of possibilities. This dilemma also extends to the question of how to integrate self-generated anomalies into existing firewall logs. In order to answer these questions, a series of experiments were carried out to provide insights and answers. Unlike much of the previous research, which often focused on improving and analyzing a limited number of algorithms, in our research, multiple algorithms were tested that include both unsupervised and supervised techniques.

The contributions of this work are as follows:

- A novel method for generating logs containing attack-related records that eliminates the need for a dedicated testbed or installed security controls.
- The ability to generate anomalies that closely resemble real-world attacker behavior, enabling seamless integration with existing firewall logs for realistic testing.
- A comparative evaluation of unsupervised and supervised machine learning algorithms in detecting injected anomalies using various feature construction, scaling, and aggregation techniques.

The structure of this paper is as follows. Section 2 presents related work that addresses the problem of anomaly detection and synthetic attack log generation. Then, Section 3 describes the firewall logs that serve as data for anomaly detection, i.e., what attributes they contain and what these attributes look like. This is followed by Section 4, which presents the proposed method for integrating attack logs into a security log and the process of generating anomalies and integrating them into our pre-existing firewall logs based on the proposed method. Section 5 describes the process of constructing relevant features from firewall log attributes for use in machine learning models. The implementation of unsupervised learning, the algorithms and performance metrics used, and the results obtained are explained in Section 6 and for supervised learning, the details are explained in Section 7. Subsequently, the limitations of the proposed method for generating anomalies and the significance of the obtained results are discussed in Section 8. Finally, in Section 9, conclusions are drawn based on the results of the experiments and ideas for future work are presented.

## 2. Related Work

Several papers have created custom, publicly available datasets for specific attack categories, including APT attack patterns [16], a variety of attacks such as DDoS, botnets, and infiltrations [10], attacks generated using the IXIA tool [17], etc.

Most methodologies propose a testbed architecture and evaluation metrics for creating valid and realistic intrusion detection system (IDS) logs [18,19,20,21,22,23,24]. What unites these studies is the common practice of artificially generating network data and logs, often involving virtual users to increase the realism of the data.

Some research proposes techniques for generating datasets, including log line clustering [25], generating network flows at fragment level [26], using Generative Adversarial Networks (GANs) to generate datasets [27], dynamically generating datasets [28], and using fuzzy association rules to integrate device logs with traffic data [29].

In contrast to the previously mentioned studies, our proposed approach to creating security logs relies on actual logs originating from the organization itself and, therefore, does not require access to a dedicated testbed or an installed security control. It is worth noting that we found only two approaches [30,31] that use authentic data from a target organization, while, in most cases, the lack of such data reduces the representativeness from the perspective of these organizations.

Roschke et al. [30] integrated IDS alert logs collected from a university network with a dataset of IDS alert logs they created by recording manually performed attacks inside vulnerable subnets. They integrated the logs in an ad hoc manner, solely to evaluate the proposed IDS alert correlation approach, without explaining or discussing the details of the method used for integrating these logs.

Maciá-Fernández et al. [31] relied on a bespoke testbed to create NetFlow logs, which they then integrated with anonymized traffic for IDS evaluation. Similarly to several previously mentioned works which also rely on specialized testbeds, this results in significantly higher resource requirements and more complex technical details that are difficult to integrate with existing traffic or event logs.

In the field of anomaly detection, numerous papers provide insight into current anomaly detection methods, highlight their respective strengths and weaknesses, and address the prevailing challenges and research gaps in this field [8,32,33,34,35]. The recent study by Nassif et al. [8] stands out as a comprehensive Systematic Literature Review (SLR) in the field of anomaly detection, aiming to summarize, clarify, and investigate the ML techniques and implementations applied in anomaly detection from 2000 to 2020. Among the 290 articles identified, 43 different applications of anomaly detection, 29 different ML models and 22 different datasets used in anomaly detection experiments were identified. The study shows that intrusion detection and network anomaly detection dominate among the other anomaly detection applications, and, among the methods, unsupervised anomaly detection methods are the predominant choice for anomaly detection. Our research has shown that the most commonly used techniques include One-Class Support Vector Machines [36], recurrent neural networks [37,38], Convolutional Neural Networks [38], Generative Adversarial Networks [7,39], Autoencoders [40], and clustering techniques [41,42]. These techniques found in the literature are in close agreement with the research of Nassif et al. [8].

Tuor et al. [43] presented a novel online unsupervised deep learning approach based on deep and recurrent neural networks to detect anomalies in the network activity from system logs in real-time. They decomposed the anomaly scores into the contributions of individual features of user behavior to increase interpretability and demonstrated the approach using the CERT Insider Threat Dataset. Some of the research in this area specifically addresses the challenge of adaptive threshold selection in unsupervised anomaly detection [44,45], while others explore ways to circumvent the need for such thresholds altogether [46].

Although unsupervised approaches have dominated the field of anomaly detection in recent years, supervised learning methods, especially when it comes to classification tasks, are still a popular research topic [8]. Two different classification tasks can be found in the literature: binary classification, where logs are classified as normal or anomalous, and multiclass classification, where logs are classified based on an action attribute that can take multiple values.

As an example of a binary classification task, Allagi and Rachh [47] applied the Self-Organizing Feature Map algorithm and K-means to identify anomalies in the access patterns with supervised ML techniques based on the publicly available dataset in the UCI ML repository, while As-Suhbani and Khamitkar [48] proposed a meta-classifier model with four binary classifiers: K-Nearest Neighbor, Naive Bayes, J48, and One R using the network log dataset.

On the other hand, Aljabri et al. [49] have classified the firewall data based on the actions *allow*, *drop*, *deny*, or *reset-both* using the different algorithms in a comparative study: K-Nearest Neighbor, Naive Bayes, J48, Random Forest, and Artificial Neural Network. Ucar and Ozhan [50] used different classification methods to detect anomalies in a firewall rule repository using the firewall logs as the data source. Shetty et al. [51] used Neural Network, Random Forest, Decision Tree, and SVM classifier to accomplish the task of intrusion detection, while Al-Haijaa and Ishtaiwia [52] used Shallow Neural Network and Optimizable Decision Tree to classify firewall data based on three action classes, *allow*, *deny*, and *drop/reset*. Lu et al. [29] integrated network device logs with network traffic data and introduced four different types of attacks to reconstruct the actions of attackers within the network, and used supervised classification learning to accomplish the task.

With the growing popularity of deep learning methods, they have also been successfully applied to the problem of classifying network data. One such example is the work of Fotiadou et al. [53], which uses Convolutional Neural Networks and Long Short-Term Memory Networks to create robust multiclass classifiers for event types based on the network logs collected by pfSense as a data source.

Furthermore, there is also an example of the combination of unsupervised anomaly detection and supervised machine learning methods [54]. Le and Zincir-Heywood [54] proposed a system that can learn from unlabeled data and a very small amount of labeled data and generalize to larger datasets to detect anomalous behaviors and unnoticed malicious actions.

In addition to traditional unsupervised and supervised machine learning techniques, the use of graphs for anomaly detection is a noteworthy topic. Harshaw et al. [55] used graphlets for anomaly detection and assumed that unusual events within the network would lead to changes in the number of graphlets. In contrast, Chae et al. [45] developed an adaptive approach for threshold selection in detection systems by representing the network as a bipartite graph. In addition, the research of Zhang et al. [56] is of interest because it uses pre-trained hidden Markov models for multistage attack detection.

There are a number of works that do not directly use machine learning in a supervised or unsupervised form, but use some sort of statistical processing or data mining techniques. Hommes et al. [57] used approaches from statistical process control and information theory to track potential incidents and detect suspicious network activity based on firewall logs provided by the ISP. Gutierrez et al. [58] employed statistical tools such as Mahalanobis distance, factor analysis, and histogram matrices to detect anomalies. They also proposed a tabular vector approach to create meaningful state vectors from time-oriented blocks, which are then analyzed with multivariate and graphical analyses.

As for the works that use data mining techniques, many of them use the WEKA data platform [59,60]. Khamitkar and As-Suhbani [60] even use a hybrid approach combining data mining and classifiers. As-Suhbani and Khamitkar [61] focused on anomaly detection in firewall policy rules. Ceci et al. [62] used a multirelational data mining approach to detect anomalies in firewall logs, which allowed data scattered in multiple relational tables to be analyzed and used to discover multivariate relational patterns. Rather than explicitly detecting anomalies, Caruso and Malerba [63] first created an adaptive model of normal daily network traffic and then detected anomalies based on the degree of deviation from the model.

Of the papers listed in the existing literature review [8] we examined those where it was stated that both unsupervised and supervised ML techniques were used and those where the type of ML technique used was not specified. Among those papers, we were able to identify three categories based on the type of task they address. The first group of papers presents a novel hybrid method for network anomaly detection by incorporating both supervised and unsupervised methods in their work [64,65,66], while the second group also presents a novel hybrid method, but also a more in-depth evaluation compared to some other methods [67]. In the third group, which focuses exclusively on the comparison between different ML techniques, a total of six papers were identified. Half of these papers compare only a small specific subset of models [68,69,70], while the other half present a larger comparative study similar to ours [71,72,73].

Van et al. [68] conducted a study comparing only two deep learning models (Stacked Autoencoder and Stacked RBM) for intrusion detection and attack classification. Similarly, Liu et al. [69] evaluated only four different clustering techniques, while Mulinka and Casas [70] compared the performance of four popular stream-based machine learning algorithms with their batch-based versions. Abdulhammed et al. [71] present the comparison of five algorithms: Deep Neural Networks (DNNs), Random Forest, voting technique (OneR, Naive Bayes, and ExtraTree), stacking technique (with Linear Discriminant Analysis, Naive Bayes, and OneR) and Variational Autoencoder. They used the imbalanced dataset CIDDS-001, which contains unidirectional NetFlow data generated in a cloud environment with OpenStack and includes 146,500 instances simulating a small business network. Normal traffic, reflecting realistic user behavior, accounts for 91.6% of network traffic. Meng [72] presents the comparison of the three models: RBFNetwork (Neural Network), SMO (Support Vector Machine) and J48 (Decision Tree). To evaluate these models, randomly selected data from the 10% KDD dataset were used, resulting in 98,804 instances, of which only 19,268 were considered normal (19.5%). The most comprehensive comparison was performed by He et al. [73], where they provided an overview of six state-of-the-art log-based anomaly detection methods, including three supervised methods: Logistic Regression, Decision Tree, and SVM, and three unsupervised methods: Log Clustering, PCA, and Invariants Mining. The selected methods were evaluated using two datasets, both from production systems and manually labeled by experts. The first HDFP dataset contains approximately 11 million log messages collected from the Amazon EC2 platform, with 0.15% labeled as anomalies. The second BGL dataset contains nearly 5 million log messages recorded by the BlueGene/L supercomputer system at Lawrence Livermore National Labs, with 7.3% of the log messages labeled as anomalous.

Nowadays, there are many toolkits that allow easier implementation of machine learning techniques. The most notable in the field of anomaly detection is *PyOD*, a Python toolbox for scalable outlier detection. Since its debut, *PyOD* has been used in various academic and commercial projects. Some examples of the use of *PyOD* include the detection of anomalies in a large-scale online pricing system at Walmart [74] and the development of an unsupervised outlier detection framework called DCSO, which has been demonstrated and evaluated for dynamically selecting the most competent base detectors [46].

In contrast to the previously mentioned studies, different machine learning models were used here, including both unsupervised and supervised approaches, to evaluate the advantages and disadvantages of these techniques for anomaly detection; while most previous research has used logs or network traffic data with pre-existing anomalies, this research used pre-existing firewall logs without anomalies and entries representing real attacker actions in the network as anomalies.

## 3. Firewall Logs

Firewall logs were used as the source dataset for our study. These logs are from a Check Point firewall deployed in the industrial control network of an electricity transmission system operator. The collected logs are structured on a daily basis, with each daily log file containing approximately twelve million records.

Log files from four different days were used for our experiments. Each log file contains communication records for a single day, and the information in each record refers exclusively to SYN segments involved in TCP protocol communication. It is worth noting that in addition to the TCP protocol, the dataset also includes the UDP and ICMP protocols.

The data originally received were already structured as a comma-separated form with 19 different fields. Due to the sensitive nature of these logs, the operator had previously anonymized them by replacing IP address ranges and identifiers in a deterministic way. Since most of these fields consist mainly of anonymized values related to the identifiers of the objects or geographical areas associated with the source and destination IP addresses and are not relevant for anomaly detection, we selected the following attributes for this investigation: connection timestamp, source and destination IP address, source and destination port, protocol attribute, and firewall action, all of which are commonly used in similar anomaly detection experiments [49].

Two days of firewall logs were used for the unsupervised learning phase. When these logs were loaded, the data records were first filtered by the firewall action attribute. This attribute can have one of four values: *accept*, *drop*, *bypass*, and *monitor*. Remarkably, accept was the predominant value and accounted for over 70% of cases, while drop occurred in about 29% of cases. The other two values had a negligible share and accounted for a total of around 170 data records. Only the data records with the action value accept were retained. The reason for this choice is that all rejected connections were classified as suspicious by the firewall and could be easily analyzed. Furthermore, as they were already blocked, they posed no real threat to the network. Even if numerous rejected connections indicate a potential attack, they can be easily identified by simple statistical observations, leading to the generation of alerts. Therefore, the use of machine learning techniques in these scenarios is considered unnecessary. Furthermore, it is important to point out that our analysis was performed under the assumption that the pre-existing logs represented the normal state of the network and thus did not contain any anomalies.

In the first phase of data analysis, one of the most important steps was to calculate descriptive statistics for the dataset. Table 1 provides an overview of the descriptive statistics for the two datasets representing two days of firewall logs used for the unsupervised learning process. Each column in the table corresponds to one of the five observed categorical attributes: source IP, source port, destination IP, destination port, and protocol. For each attribute, the following statistics are presented: the number of values (Count), the number of unique attribute values (Unique), the most frequent attribute value (Top), and the frequency of occurrence of the most frequent attribute value (Freq). It is worth noting that the statistics for the timestamp attribute are not shown in this table, as the values for this attribute were usually evenly distributed throughout the day.

In addition to the basic descriptive statistics, a comprehensive analysis of the values within each attribute and their respective distributions was carried out. For the timestamp attribute, which indicates when the connection was established, the values were usually evenly distributed throughout the day. However, there were cases where usage was subject to slight fluctuations, resulting in slightly higher or lower activity.

An examination of IP address attributes revealed that there were around 2000 to 2500 different IP addresses every day. This range of IP addresses is to be expected for a large industrial network. The distribution of destination IP addresses appeared to be fairly uniform. In contrast, the distribution of source IP addresses showed remarkable variation. One particular IP address, 143.198.132.18, was responsible for about 15% to 20% of all connections, depending on the day. Other source IP addresses rarely exceeded a 5% share of connections.

The values in the source and destination port attributes appear numeric, but are actually categorical because of the limited relationship between the numbers. The source port attribute included over 63,000 different values. The most common values were 99,999 (about 7%, a consequence of the ICMP protocol) and 123 (about 3% of all connections). Of the other source port values, only 2304, 2305, and 60,076 occurred in more than 0.1% of connections each. In contrast, the destination port attribute contained a manageable set of about 1600 unique values. The most common destination ports included 53 (about 21%), 443 (about 13%), 80 (about 10%), 123 (7%), 99,999 (7%), 88 (5%), 2404 (4%), 8080 (3%), and 389 (3%). All other destination ports accounted for less than 2% of connections each.

The protocol attribute offers three different values: *tcp*, *udp*, and *icmp*. Most connections were categorized as tcp (about 59%), followed by udp (about 34%), with icmp making up the smallest percentage (about 7%).

## 4. Generating Synthetic Logs

This section presents an approach for developing customized methods to integrate different types of artifacts into logs. The process of generating logs to represent anomalies in our existing logs is described in detail, along with an explanation of how these anomalies were integrated into the pre-existing logs.

### 4.1. Methodology

**Figure 2:**
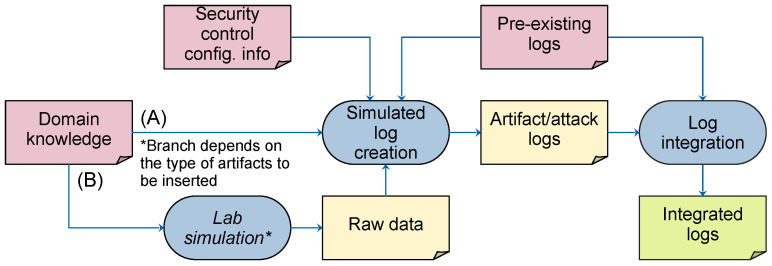
The proposed method for log synthesis. Based on the type of attack artifacts to be created, domain knowledge is used to create logs that contain artifacts to be inserted, which are then combined with the organization’s existing logs to create the final synthetic logs, as explained in Section 4.

In Figure 2, the proposed method is presented, which can serve as a basic framework for developing tailored methods for incorporating various types of artifacts into logs, including those related to cyberattack techniques [2]. Rounded rectangles represent the processes of the method, while its inputs and outputs are represented by the document symbols and connected by arrows. The required inputs for this method include the following:

- *Domain knowledge describing target attacks and security controls*. This input includes various information, including: (i) reports of attacks and their consequences, such as threat or malware reports and pre-recorded network traffic samples, (ii) knowledge of the security control for which the logs are synthesized, and (iii) knowledge of the medium through which the artifacts are manifested. For example, when creating artifacts originating from network scans for firewall logs, knowledge of the network scanning tools used, such as nmap [75], knowledge of the firewalls used, and familiarity with network protocols are required.
- *Security control configuration information*. This information can be obtained in a variety of ways, such as by consulting documentation, talking to the relevant security administrators, or directly accessing the configuration of the security control in question. This paper assumes that the security control is configured according to the reports provided and its equivalence is not questioned. For example, in the case of a network firewall, this information could include policy descriptions in unstructured text obtained from discussions with administrators, firewall policy documentation, or a set of configured firewall rules. Depending on the level of detail desired, this information can even be gathered verbally through interviews with the appropriate personnel.
- *Pre-existing logs*. These logs represent authentic data collected by security controls within the organization. Most organizations already maintain logs for audit purposes, so they are readily available in practice.

Once the input data had been collected, the first step was to determine whether the domain knowledge collected was suitable for log generation. If the input data proved to be insufficient, they could be used to perform a laboratory simulation, as shown by branch (B) in Figure 2. This simulation aimed to obtain additional information that included all inputs used by the security control in question for log generation. In cases where the input data were already comprehensive, this step was bypassed and the method proceeded with the creation of simulated logs, as shown by branch (A).

Readers might notice that parts of the method as shown in Figure 2 are not strictly formally defined, with only the fundamental requirements of its components described. This is intentional. Due to the inherent differences between logs of various security controls and attacks, the method aims to define the approach using high-level guidelines rather than formal definitions and implementation details. Implementations for concrete security control logs may introduce additional specific formal and technical considerations; but, based on our research, we expect such additions to be refinements of the method specific to those security control logs, not necessarily applicable to other types of logs.

To illustrate, let us consider the goal of creating network firewall logs in the context of the malware *M*. Identifying the necessary information begins with an analysis of how firewalls create logs. Firewalls create logs based on network traffic and process elements such as IP addresses, protocols, and ports. Stateful firewalls can also track communication sequences.

In cases where a sample of the malware *M* is available and it can be isolated and executed in a controlled environment (sandbox), its characteristic network traffic can be recorded and used as a source of domain knowledge. If no sample of the malware *M* is available, its behavior can be reconstructed from reports describing the inner workings of the malware *M*, especially if these reports contain descriptions of its command and control (C2) communications and exploitation mechanisms. However, if no such reports or samples exist, you will need to obtain data about the malware through a laboratory experiment. This may involve activities such as malware reverse engineering or running the malware *M* in a sandbox.

The second step involves the creation of attack logs through simulation. This is the stage where the specific methods derived from the proposed method may differ depending on the type of artifacts and the level of detail required. Some types of artifacts and attack logs can be created manually, while others require code development to simulate more complex behaviors. In this step, it is important to analyze the pre-existing logs to understand what needs to be tracked when creating the artifact/attack logs. This analysis also helps determine the format and conventions that need to be followed to ensure that these logs have common fields and can be integrated seamlessly.

The final step is to integrate the created artifact/attack logs with the organization’s pre-existing logs to obtain the final integrated logs. This step depends heavily on the semantics of the logs and can include tasks such as setting correct sequence numbers, assigning meaningful timestamps, and ensuring that the data make sense from a domain perspective. For example, if the goal is to create a log that includes a malware installation, it is important to configure timestamps, IP addresses, and ports to accurately reflect the attacker’s actions, including lateral movement and malware installation on workstations. Realistic sequences and timing should be maintained.

### 4.2. Generated Anomalies

In this study, as described in Section 3, the firewall logs only captured connections that complied with the configured policies and did not violate them. To overcome this limitation, the proposed method was validated by creating multiple sets of attack logs and seamlessly integrating them with the pre-existing logs from the real network of a critical infrastructure operator. In addition to the pre-existing logs, a comprehensive description of the network’s topology and firewall configuration was obtained through a series of interviews with an administrator. Furthermore, a list of potentially interesting attacks was compiled by considering the possible attacker targets and attack techniques manifested in the logs, the detection of which would have the potential to block subsequent attacks. It is important to clarify that the purpose of this paper is not to create a threat model for the target system or a comprehensive catalog of attack techniques, as these aspects are beyond the scope of this paper.

Logs with different types of network scans and command and control (C2) communication were selected for demonstration purposes. Some of these techniques required simulation to generate data, while others required manual data generation. The following is a comprehensive list of all the techniques for which logs were created:

1. *SYN scans of a machine and a target range of machines using nmap*. These network scans were created by scanning our local network server and local IP range with the *nmap* [75] tool installed on a Kali Linux [76] virtual machine (VM). Both tests were run with the nmap flags −sS. Since the firewall only logged initial packets for individual ports and did not log return packets, it was not necessary to set up listeners for real applications.
2. *Connect scans of a machine and a target range of machines using nmap*. This was done similarly to the first scan, except that the −sT flags were used.
3. *UDP scans of a machine and a target range of machines using nmap*. This was done similarly to the first scan, except that the −sU flags were used.
4. *SYN scan for characteristic VPN services using nmap*. This scan was performed in a similar way as the first scan, but only with the entire IP range and ports 102, 6001, and 13,777.
5. *Connect scan for characteristic VPN services using nmap*. This was done similarly to the fourth scan, except that the flags −sT were used.
6. *Establishment of an RDP session, with several unsucessful atempts*. This was simulated by setting up a Windows 10 VM and making several attempts to establish an RDP connection. The first connections were made with an incorrect password, the last with the correct password.

The network traffic resulting from the above scans was recorded using Wireshark on the Kali Linux VM. We deliberately chose to run these scans instead of creating the logs manually to capture the realistic timing of the packets. A script available in [77] was developed and used to convert the packet logs into logs compatible with the target firewall logs. The lab environment in which the network scans were collected consisted of a virtual machine running Kali Linux and several specified scan targets. Both individual targets and the entire subnet were scanned using the *nmap* tool [75]. Then, network traffic generated during these scans was recorded using *Wireshark*. Various combinations of ports common to the target network were scanned using different nmap scan types, including SYN, TCP connect, and UDP scan. Since our firewall logs provide significantly less detailed information compared to full packet captures (PCAPs), a thorough manual inspection of all attributes was performed in the generated anomalous logs. This careful inspection was critical to ensure that these logs accurately reflected what would be expected in the event of an actual attack on the target network.

The integration process began with the selection of IP addresses from the pre-existing logs. The attack logs were then seamlessly inserted between these addresses at specific timestamps to ensure that the timing of the packets remained realistic. When selecting IP addresses, it is important to consider the context of the applications and services associated with those addresses, as well as the number of mutual connections. The level of stealthiness desired by the attacker is directly related to the contextualization of the logs. For example, if an attacker wants to perform a stealthy network scan from an infected device, he would carefully send packets to devices and subnets that the infected device normally communicates with. Any unusual behavior, such as connecting to a service for the first time or connecting outside of working hours, would generate a suspicious log entry. This contextualization is a key factor in creating logs that closely resemble real attack scenarios.

The described integration process was performed using a developed script. After capturing the raw network traffic, it was converted to the format of the target firewall logs using a script that mimics the data captured by the target firewall control to create attack logs. The script filtered out irrelevant parts of the traffic, identified the first packets exchanged between two endpoints, assigned the corresponding date and time to the first event, and replaced individual addresses or IP address ranges with the specified targeted addresses. It is worth noting that this step can vary greatly depending on the configuration of the target security control. The result of this conversion was a set of attack logs fully prepared for integration with the pre-existing logs.

To integrate these attack logs with the pre-existing logs, they were simply inserted and the entire dataset was organized based on the timestamp. To distinguish between the records from the pre-existing firewall logs and the generated attack logs, a label attribute was introduced. The value of this attribute was set to zero for records from the pre-existing logs and to one for the attack logs that are considered anomalies.

The final integrated logs were now ready to serve as a dataset for various tasks. The generated anomalies could be used in two ways as part of anomaly detection. One approach involved an evaluation step in unsupervised learning, where an unsupervised machine learning model was applied to an unlabeled dataset consisting of a mixture of pre-existing logs and generated anomalies. This model assigns each record a score indicating the extent to which it is anomalous according to the model. The performance of the model could then be evaluated based on its ability to effectively distinguish between pre-existing logs and the generated anomalies. Alternatively, it was possible to use supervised learning, in which a supervised machine learning model was trained on a labeled dataset that combines pre-existing logs and generated anomalies. The model was then used to predict labels for an unlabeled dataset that is a mixture of pre-existing logs and generated anomalies.

## 5. Feature Construction

In order to use most machine learning algorithms, it is essential to create a dataset that consists solely of numerical values. However, our current dataset, which consisted mostly of categorical string values obtained from firewall logs, had to be converted to numeric representations.

There are two main methods for converting strings to numeric values, each with its own advantages and disadvantages. The first method involves a direct conversion when the string already represents a numeric value. However, this approach may not account for cases where the values are categorical, potentially introducing unintended rank and distance information that was not present in the original data.

The second method, on the other hand, uses one-hot coding, which is widely used for categorical data. However, it has the disadvantage of driving up computational costs, since one-hot coding generates a set of new attributes equal to the number of distinct values within an attribute.

Considering the aforementioned factors, this study explored different attribute transformation options. For the timestamp attribute, the strategy of direct conversion to a large integer and further division into two additional features, namely *hour* and *time*, was used. The hour feature represents the hour of the day the connection was initiated in integer format, while time corresponds to an integer value of the timestamp without a date.

For the IP address attributes, different transformation methods were investigated. First, direct conversion to integers using the Python package *ipaddress* was considered. Alternatively, an attempt was made to divide the IP address into four integers and create four different features based on this segmentation. The third approach was to convert each of these four integers into 8-bit binary numbers, resulting in a total of 32 newly constructed binary features.

Given our data analysis, which revealed that IP address attributes can include up to 2500 different values, traditional one-hot encoding was deemed impractical. Therefore, our last strategy for IP address transformation was a hybrid approach. First, the IP address was split into four integers and, then, one-hot encoding was applied to each of these four new attributes, resulting in a comprehensive zero–one vector.

For attributes describing source and destination ports, it was easiest to retain their original integer representation. It is important to note that all connections running over the ICMP protocol had the port value *unknown*, and, for these, the value 99,999 was assigned. One-hot encoding was another viable option that, according to our preliminary analysis, was particularly suitable for the destination port attribute due to the number of distinct values. Two hybrid approaches were implemented in this context:

- The first method used one-hot encoding exclusively for “important ports”, which are defined as ports that occur in more than 1% of all connections. For all other ports, an additional feature called *other* was introduced, resulting in a significantly reduced zero–one vector.
- The second approach categorized ports into ephemeral and non-ephemeral categories. Ports with numbers lower than 1024 were encoded with the one-hot encoding, while ports with higher numbers were represented with the *other* feature. This approach resulted in a 1024-bit zero-one vector.

Finally, one-hot encoding was used for the protocol attribute, as it only contained three different values.

After the construction of the features, the features were scaled using three different methods: standard scaling, min–max scaling and robust scaling. In standard scaling, the mean value of the feature is subtracted from its value and the result is divided by the standard deviation. In min–max scaling, the feature values are transformed to fit the range [0, 1]. In the robust scaling method, each feature value is adjusted by subtracting the median and the data are scaled based on the interquartile range.

In addition to using raw firewall logs, aggregated logs were also used to test anomaly detection models. Aggregation was performed at three different levels. At the first level, connections were grouped based on attributes: hour, IP addresses, ports, and protocol. At the second level, the grouping was done based on the same attributes, but without the source port. Finally, at the third level, both source and destination ports were omitted from the grouping.

Several new features were introduced for each aggregation level. The first level contained the feature *hits*, which indicates the number of connections within each group. At the second level, in addition to the features added at the first aggregation level, features indicating the standard deviation of source ports, the most frequent source port, and the number of unique source ports in each group were added. Finally, at the third level, the same features as at the second level of aggregation were added, in addition to features for the standard deviation of the destination port, the most frequent destination port, and the number of unique destination ports in each group. This approach resulted in a significant reduction in the number of records by a factor of about 3, 60, or 75, depending on the level of aggregation chosen.

Since we initially received the data in the form of comma-separated values, it was not possible to use additional data mining or deep learning techniques to extract additional features from the obtained firewall log data. Furthermore, since the number of available features was relatively small, we went through and tested each subset of features individually, which is presented later in Section 6.3.3. For this reason, the use of advanced feature extraction and feature selection methods is beyond the scope of this paper.

## 6. Unsupervised Learning

This section provides the definitions for each performance measure used in this paper. It also describes the methodology for performing unsupervised learning and presents the results obtained using this methodology to detect anomalies in different configurations of our dataset.

### 6.1. Performance Measures

The anomaly detection problem can be seen as a classical binary classification problem. Here there are two classes 0 and 1. Class 0 stands for normal data and class 1 for anomalous data. Two crucial metrics in classification discussions are precision and recall values. Precision represents the proportion of positive samples that were correctly classified relative to the total number of positive predicted samples, while recall represents the ratio of positive, correctly classified samples to the total number of positive samples [78]. The main problem here is that the results of anomaly detection usually depend on the threshold used. If the threshold value is increased, the precision increases, but the recall metric decreases. Recall should be optimized if it is more important to detect all true positives (anomalies) than to generate a low number of false alarms. On the other hand, precision should be optimized if the detection of false alarms is costly and it is, therefore, worth considering all positive predictions.

Since optimization by precision or recall alone can lead to the selection of a suboptimal model, many papers in this area use metrics that combine precision and recall. One of the best-known measures that combines precision and recall in one measure is the F-beta measure, from which a variety of measures can be derived depending on the desired importance of precision and recall. One of the best-known measures is the *F*1-*score*, which is often used in classification problems. This measure represents the harmonic mean between precision and recall, i.e., it attaches equal importance to precision and recall and can be calculated using the following formula:

(1) $$\begin{array}{c} F1-\mathrm{score}=\frac{2\times precision\times recall}{precision+recall} \end{array}$$

Another measure from the family of F-beta measures that is also frequently used is the *F*2-*score*. Here, recall is weighted more heavily than precision. This is particularly useful when detecting anomalies in network data, as it is most important in critical systems to detect all anomalous events and then reduce the number of false positives as much as possible. The *F*2-*score* is defined as follows:

(2) $$\begin{array}{c} F2-\mathrm{score}=\frac{5\times precision\times recall}{4\times precision+recall} \end{array}$$

All the above measures depend on the chosen threshold. Sometimes it is difficult to find a good method to select the threshold, especially if this is to be done in an unsupervised way. For this reason, there are some measures that are independent of the chosen threshold and can be used to compare different models for unsupervised anomaly detection. The first approach is to use the Receiver Operating Characteristic (ROC) curve. The ROC curve visualizes the trade-off between recall and false positive rate (FPR). In other words, it is a plot of the true positive rate (TPR) as a function of the false positive rate (FPR) for each possible threshold [79]. To determine which ROC curve is better than another, the area under the ROC curve (AUC ROC value) can be calculated. The larger the AUC ROC value, the better the model, regardless of the selected threshold value [80]. The values of the AUC ROC range from 0, if the model always predicts incorrectly, to 1, if the model is perfect and predicts everything correctly. The base value here is 0.5, which can be used to determine how well the model predicts [81]. The second commonly used approach is the one that uses the precision-recall (PR) curve. The PR curve is a plot of precision as a function of recall [82]. Similar to the ROC curve approach, the area under the PR curve (AUC PR score) can be calculated to obtain a number that describes the performance of the model and also ranges from 0 to 1. In contrast to the baseline of the AUC ROC score, the baseline here is based on the number of samples in each class and is not fixed as with the AUC ROC. The baseline of the AUC PRC is determined by the ratio of positives ($N_{p}$) and negatives ($N_{n}$) as $N_{p}/\left(N_{p}+N_{n}\right)$ [81].

When comparing the two approaches described, it is important to note that the AUC ROC score is only suitable if the data classes are balanced and if positive and negative classes are equally important. The AUC PR score, on the other hand, is more suitable if the data are very unbalanced and the positive class is more important than the negative one. When detecting anomalies in network data, there are many more normal data than anomalous data, so the classes are often very unbalanced. Furthermore, it is more important to detect all anomalies first and then minimize the number of false positives. Therefore, optimization of the AUC PR score should be preferred to optimization of the AUC ROC score when detecting anomalies.

### 6.2. Methodology

Our anomaly detection system was parameterized with two inputs: a model (algorithm) and a subset of features used. On the first day of the firewall logs, the system initialized the unsupervised learning model, retrieved the anomaly score for each record in the dataset, and set the threshold for anomalies based on these scores. On the second day of the firewall logs, the anomaly score was calculated for each record using the already initialized model from the previous step. Based on these scores, the predicted label was zero if it was below the threshold and one if it was above the threshold. The classification metrics were then calculated based on the predicted values and the values of the *label* feature. It is important to note that the feature label was extracted at the beginning and was only used to calculate the classification metrics.

We implemented two different methods to calculate the threshold for anomaly scores. The first method was based on the PR curve. First, pairs of precision-recall values were calculated for each possible threshold, leading to different classifications. Then, the *F*1-*score* was calculated for each pair, effectively giving us an *F*1-*score* for different thresholds. The appropriate threshold value for which the *F*1-*score* was maximum was then selected. The second method was based on the fact that when detecting anomalies, it was most important for us to detect all or almost all anomalous connections while minimizing the number of false positives. For this reason, a customized threshold selection method based on the PR curve was developed to achieve a very high recall value. Here, too, the threshold value was calculated on the basis of the PR curve, but only threshold values for which the recall was greater than 0.95 were taken into account. The threshold value with the best precision measure was selected from this group of threshold values. Essentially, the threshold was selected where the recall value was greater than 0.95 and the precision value was maximized.

As already mentioned, the test method used two days of firewall logs in which anomalies had been inserted. In Table 2, you will find basic statistics on the number of records for each of the two days of logs used and for each level of aggregation implemented. The table shows the total number of records in each dataset, as well as the number of records that came from pre-existing firewall logs (normal data) and the number of records that were injected (anomalous data). From Table 2, it can be seen that the percentage of anomalies is very low, which is to be expected when detecting anomalies in network data. For unaggregated data, there are, on average, only about 0.028% anomalies in the datasets. At the first aggregation level, this percentage increases to 0.063% and, at the second aggregation level, to 1.21%, while at the third aggregation level, the percentage of anomalies is about 0.55%.

For unsupervised anomaly detection, many algorithms were originally considered for testing, many of which are commonly used in anomaly detection applications [8]. Although unsupervised algorithms such as the Connectivity-Based Outlier Factor [83], fast outlier detection using the Local Correlation Integral [84], and Stochastic Outlier Selection [85] were originally considered, they were not included in our experiments due to their space (memory) complexity. The initial study showed that their use with our dataset required the allocation of more than half a terabyte of memory, depending on the algorithm, which was not feasible. Due to the large dataset used in this study, algorithms such as One-Class Support Vector Machines [86], Fully connected Autoencoder [87], and Variational Autoencoder [88] could also not be used in our experiments due to the very long training time. On the other hand, algorithms such as Deep One-Class Classification [89] and Single-Objective Generative Adversarial Active Learning [90] were found to be unusable in a smaller analysis presented in [2], as they yielded much lower *F*1-*score* on the test dataset based on the same firewall logs as in this study, which is why their results are not discussed in this study.

In the end, a total of 13 different unsupervised machine learning models were tested, namely Unsupervised Outlier Detection Using Empirical Cumulative Distribution Functions (ECOD) [91], Copula-Based Outlier Detection (COPOD) [92], Rapid distance-based outlier detection via sampling (Sampling) [93], Principal Component Analysis (PCA) [94], Minimum Covariance Determinant (MCD) [95], Clustering-Based Local Outlier Factor (CBLOF) [96], k Nearest Neighbors (kNN) [97], Histogram-Based Outlier Score (HBOS) [98], Isolation Forest [99], Lightweight On-line Detector of Anomalies (LODA) [100], Local Outlier Factor (LOF) [101], Outlier Detection with Kernel Density Functions (KDE) [102], and Feature Bagging [103]. The implementation of the above unsupervised learning was supported by the open-source Python toolbox *PyOD*, which is used to detect anomalies in multivariate data [104].

### 6.3. Results

To test unsupervised learning for anomaly detection using firewall logs, four different types of experiments were conducted, namely what results were obtained when using different unsupervised models for anomaly detection, what results were obtained when selecting different feature construction methods, what results were obtained when selecting different subsets of features, and what results were obtained when using different scaling methods.

#### 6.3.1. Comparison of Unsupervised Models

The first question was which of the unsupervised models provided the best results for our dataset. A complete subset of features was selected for this purpose, including timestamp, hour, time, source and destination IP address, source and destination port and protocol, the simplest feature construction method, and the min–max scaling method. The simplest feature construction method was the one in which each attribute was directly converted into a numeric format, except for the protocol attribute, for which one-hot encoding was used.

**Figure 3:**
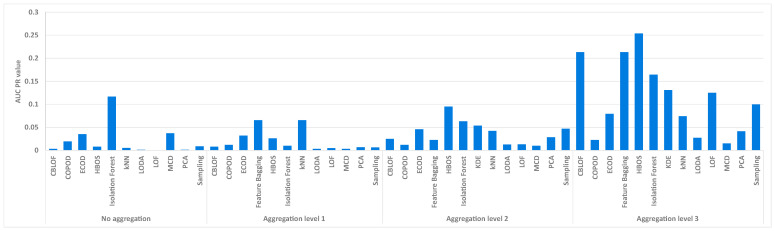
Comparison of the average AUC PR value over two days of firewall logs obtained with 13 different unsupervised machine learning models at four different aggregation levels.

First, the results are presented independently of the threshold, i.e., without selecting the threshold, which is possible by calculating the AUC ROC and AUC PR values. The AUC PR value was chosen as the measure because, as already explained, it is more suitable for datasets with unbalanced classes. A total of 13 different unsupervised models were tested at four different aggregation levels. Since some of the models did not produce the same result every time they were run, each model was run five times and the average performance values were collected over these runs. Figure 3 shows the average AUC PR value averaged over five executions for two days of firewall logs. Some of the models could not be run at a certain aggregation level in a reasonable amount of time, so their results were not included. Of the selected models, Isolation Forest provides the best result, with an average AUC PR of 0.11 without any type of aggregation, while all other models fall below the 0.05 value. At the first level of aggregation, only the kNN model achieves a value above 0.05, with an average AUC PR of 0.06. At the second level of aggregation, the models perform slightly better overall, but only the HBOS model comes close to the average AUC PR of 0.1. At the third level of aggregation, the models perform best overall, and the best is HBOS again, with an average AUC PR of around 0.25, while CBLOF and Feature Bagging also achieve an AUC PR of over 0.2.

**Figure 4:**
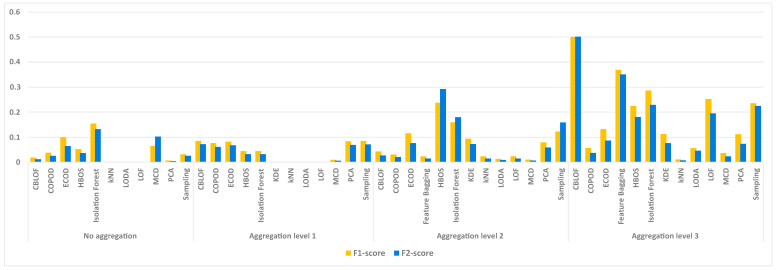
Comparison of the average *F*1-*score* and *F*2-*score* on the second day of firewall logs obtained with 13 different unsupervised machine learning models at four different aggregation levels.

After identifying the best performing model regardless of the chosen threshold, the next step is to present the average *F*1-*score* and *F*2-*score* values from five execution runs, focusing specifically on the second day of the firewall logs. These results can be seen in Figure 4. As before, the results of the models that could not be executed at a certain aggregation level in a reasonable time are not shown. Isolation Forest provides both the best *F*1-*score* and the best *F*2-*score*, with values of 0.15 and 0.13, respectively. At the first aggregation level, the results are worse and no model achieves an *F*1-*score* or *F*2-*score* close to 0.1. At the second aggregation level, HBOS and Isolation Forest provide the best results, with *F*1-*score* of 0.24 and 0.16, respectively. At the same time, these models also achieve *F*2-*score* of 0.29 and 0.18. At the third aggregation level, almost all models achieve significantly higher *F*1-*score* and *F*2-*score*. CBLOF and Feature Bagging stand out from the others at this aggregation level. CBLOF achieves a value of around 0.5 for both the *F*1-*score* and *F*2-*score*, while the Feature Bagging model achieves a value of around 0.36.

**Figure 5:**
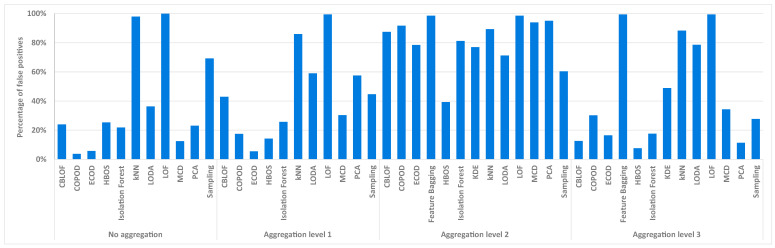
Comparison of the average percentage of false positives over two days of firewall logs obtained with 13 different unsupervised machine learning models at four different aggregation levels.

The previously presented results were obtained by calculating a threshold to maximize the *F*1-*score*. In addition, performance was considered based on the threshold obtained by optimizing precision, with a recall value of at least 0.95. Again, all selected models were tested at four aggregation levels. This time, it was not the *F*1-*score* or *F*2-*score* that were compared, but the percentage of false positives out of the total number of connections, defined as $\frac{FP}{TP+FP+TN+FN}\times 100\%$. This measure was chosen to get a sense of how much additional manual work is required for a security analyst to detect anomalies. The measure averaged over two days for selected models is shown in Figure 5. The figure shows that, on average, the best results are achieved without aggregation, with about 38% false positives. Of the selected models, the COPOD model without aggregation gives the best results, with about 3% false positives, which does not sound like much, but in absolute numbers, this is close to the 300,000 connections in a single day of firewall logs that would need to be manually checked. At the first level of aggregation, the ECOD model performs best, with around 4% false positives. At the second aggregation level, all tested models perform poorly, while at the third aggregation level, the HBOS model performs best, with around 8% false positives.

**Figure 6:**
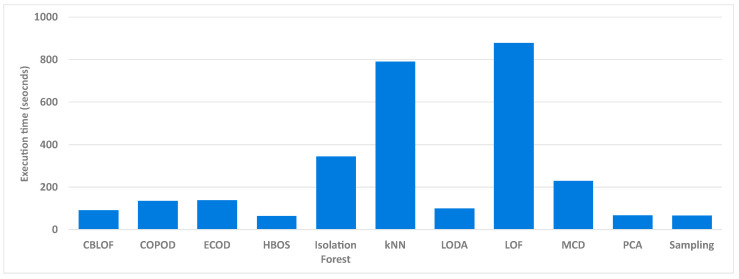
Comparison of the average execution time of the test methodology in seconds obtained with 11 different unsupervised machine learning models with no aggregation applied.

An important aspect when comparing the models for detecting anomalies is the execution time of the individual model. The time required by the entire process described in Section 6.2 was measured. Only the execution times for the data without aggregation are shown here, as the relative difference between the execution times of the models remains approximately the same. Figure 6 shows the execution times in seconds of 11 unsupervised models. The Feature Bagging and KDE models are not listed here, as they could not be executed in a reasonable time at this level of aggregation. The execution times shown indicate that the LOF and kNN models require 880 and 790 s, respectively, and are therefore the slowest. Only two other models take more than 200 s to execute, namely the Isolation Forest model and MCD. On the other hand, the PCA, HBOS, and Sampling models are the fastest, performing the test methodology in just one minute.

When comparing the execution times between the aggregation levels, a reduction of around 60% was observed when switching to the first aggregation level. When moving to the second aggregation level, the execution time was reduced even more, namely by almost 100% compared to the execution time of the first aggregation level. When choosing the third aggregation level, on the other hand, the execution time was not significantly reduced, only by around 1–2% on average compared to the second aggregation level.

#### 6.3.2. Comparison of Feature Construction Methods

The next experiment consisted of comparing different feature construction methods on the same model. For this purpose, the ECOD model was chosen for its deterministic behavior, stability, and very fast performance. Min–max scaling was again chosen as the scaling method and the following subset of attributes were selected: timestamp, source and destination IP address, source and destination port, and protocol. From the results of our tests, it was concluded that the direct conversion of the IP address into a numeric form provided a better result than the division of the IP address into four numbers, while the other two conversion methods could not be performed due to an excessive increase in the occupied memory. When converting the ports, a comparison was made between keeping the ports in numeric form and using one-hot encoding for non-ephemeral and important ports, while pure one-hot encoding was no longer feasible due to the increase in occupied memory. In the end, leaving the ports in purely numerical form provided the best results. Since the number of different protocol values was very small, one-hot encoding was easy to implement and only this conversion was considered for the protocol attribute.

#### 6.3.3. Comparison of Feature Subsets

In the subsequent experiment, the focus was on investigating how the selection of different subsets of features influenced the results obtained. The ECOD model was chosen as the reference model. As with the model comparison experiment, the simplest method was used for the feature construction and the min–max method for the scaling method. This experiment was conducted using only the raw firewall logs, without any aggregation.

In this phase, all available subsets of features were thoroughly tested and evaluated. There were a total of eight features to choose from, namely: timestamp, hour, time, source_ip, source_port, destination_ip, destination_port, and three features from the zero–hot encoding of the protocol attribute, which were observed as a group called protocols. In addition to these features, the label feature was always present in all subsets. This experimental setup resulted in 255 different subsets. All these subsets were evaluated using the average *F*1-*score* obtained for the firewall logs of two days.

**Figure 7:**
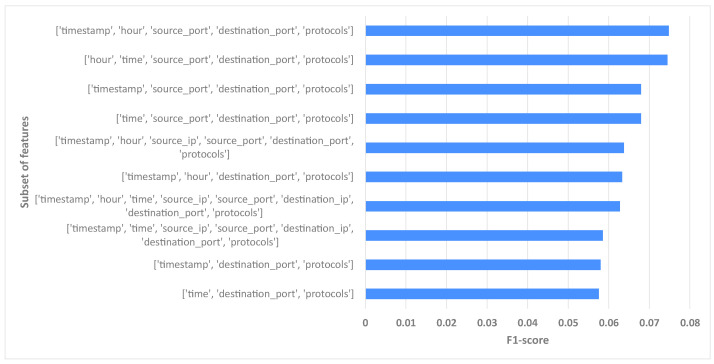
Comparison of the 10 best average *F*1-*scores* over two days of firewall logs obtained by using 255 different subsets of features, together with the ECOD unsupervised machine learning model without aggregation.

Since there are a large number of different subsets to be presented here, only the 10 best subsets are shown in Figure 7. The results show that the best subset is the one that contains the features timestamp, hour, source_port, destination_port, and protocols. The given feature group results in an average *F*1-*score* of 0.075.

To find out which features were most important, the average rank of the subsets containing each feature was calculated (with the best subset having a rank of 1 and the worst a rank of 255). Accordingly, the features timestamp, time, and protocols had the lowest average rank and were, therefore, considered the most informative. The feature $destination_{i}p$, on the other hand, had by far the highest average rank and was, therefore, considered the least important feature based on this experiment.

To further analyze the relationship between the individual features, the Pearson correlation coefficient between each pair of features was calculated. The Pearson correlation coefficient is a correlation coefficient that measures the linear correlation between two variables and is calculated as the ratio between the covariance of two variables and the product of their standard deviations [105]. This coefficient is defined as a number between −1 and 1 and is interpreted as a measure of the strength and direction of the relationship between two variables.

**Figure 8:**
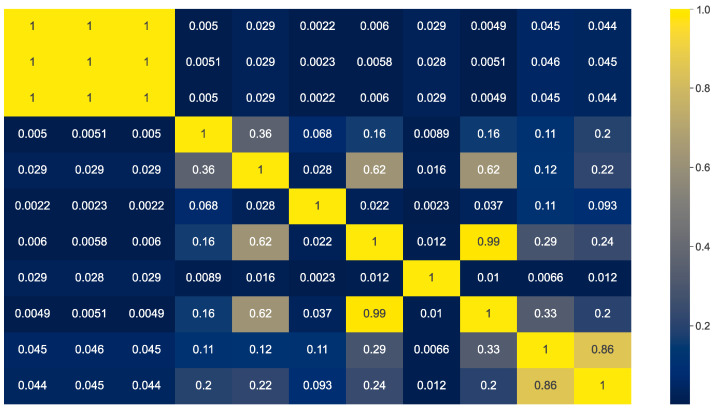
The heatmap of the Pearson correlation coefficients for each selected pair of features.

The heatmap of the calculated correlation coefficients is shown in Figure 8. The heatmap shows that the correlation between the features timestamp, hour, and time has a value of 1, which means that they are perfectly positively correlated. There is also an almost perfect positive correlation between the feature destination_ip and the feature protocol_icmp (part of the zero–one vector). A strong positive correlation is observed between protocol_tcp and protocol_udp, with a coefficient of 0.68. There is a moderate positive correlation between the features source_port and destination_port, as well as protocol_tcp and protocol_icmp. The correlation between all other pairs of features is weakly positive or almost non-existent.

We used the calculated Pearson correlation coefficients to reduce the number of features with the aim of removing all highly correlated features. This was done in an iterative procedure, so that one feature was removed in each iteration. In each iteration, the Pearson correlation coefficient was calculated for each remaining pair of features. The pair with the highest correlation coefficient and a value greater than 0.8 was selected for elimination and one feature from this pair was removed. The procedure was repeated until no more features are removed.

Based on the implemented procedure, the reduced set of features included the following features: timestamp, source_ip, source_port, destination_ip, destionation_port, protocol_tcp, and protocol_udp (including the feature label). This set of features was used according to the same methodology as before in the evaluation of the different subsets. The results obtained with this subset of features yielded an average *F*1-*score* of 0.012. When adding this result to the already existing results for the subsets of features, a rank of 186 was obtained out of 256 different subsets. This shows that the subset obtained by removing the correlation provided a result that was close to the average results of all the different subsets of features.

#### 6.3.4. Comparison of Scaling Methods

Finally, we examined how a change in the scaling method used affected the results. In the experiments conducted, it was found that the scaling method only affected the results for the second day of the firewall logs. Of the three scaling methods tested, min–max scaling proved to be the best, followed by robust scaling, while the standard scaling method was the worst.

## 7. Supervised Learning

From the related work presented in Section 2, two different types of classification tasks have emerged: one is binary classification, in which firewall logs are classified as normal or anomalous, and the other is multiclass classification, in which firewall logs are classified based on a firewall action attribute. As we explained in Section 3, the firewall logs were first filtered by the firewall action attribute, so that only connections with the value *accept* were retained. Therefore, the task in this study was not multiclass classification. Instead, we integrated the anomalous records into the pre-existing firewall logs and introduced a feature label that distinguishes between normal and anomalous records. In this way, we have transformed the problem of unsupervised anomaly detection into the task of binary classification, where we can apply supervised machine learning methods. The following section describes the methodology we used for our supervised learning experiments and presents the results obtained.

### 7.1. Methodology

In supervised learning experiments, the following approach was used to transform features: IP addresses were directly converted to large numeric representations and one-hot encoding was applied to the protocol attribute, while the other attributes were left in their original numeric form.

During the training phase, two days of pre-existing logs were merged with one day of logs with integrated anomalies. Six-fold cross-validation was used to find the optimal model for the training dataset. In *k*-fold cross-validation, the set of observations is randomly divided into approximately equal *k* groups (folds). The first fold is treated as the validation set, and the supervised model is fit on the remaining *k* − 1 folds [106].

After training the model, the trained model was used to make predictions for an unlabeled day of firewall logs with anomalies inserted. The evaluation of the performance of our model was based on the comparison of the predicted labels with the actual labels, and the *F*1-*score* was used as the primary performance measure.

### 7.2. Results

The procedure described above was used in the test phase. A total of 13 different supervised learning models were evaluated, including Naive Bayes, Kernel Naive Bayes, Linear Discriminant Analysis, Quadratic Discriminant Analysis, Regularized Discriminant Analysis, Rule Induction, Logistic Regression, Random Forest, Decision Tree, Decision Stump, Random Tree, Gradient Boosted Trees, and Perceptron. In these tests, it was found that the inclusion of IP addresses in the training of the models led to overfitting. Therefore, features describing IP addresses were not included in the supervised learning experiments.

When reviewing the test results, some models were found to have an *unknown F*1-*score*, which was due to the fact that these models predicted all given records as normal. These models were not included in further analysis. To analyze the performance of the selected models, the following performance measures are presented: recall, precision, *F*1-*score*, and *F*2-*score*. The above measures for the six selected supervised models at the second and third levels of aggregation are presented in Table 3. The first level of aggregation and the unaggregated data were not included in this analysis due to the high resource requirements.

Based on the *F*1-*scores* and *F*2-*scores* from Table 3, it can be seen that all models perform better at the second aggregation level, with the exception of the Naive Bayes model. When moving from the second to the third level of aggregation, there is a shift in the recall and precision values, so that the recall values generally decrease, while the precision values increase, which is due to an increasing percentage of false negatives. At the second level of aggregation, all models except Naive Bayes perform excellently, achieving an *F*1-*score* of about 0.9. Of the models tested, the Kernel Naive Bayes performs best at the second level of aggregation, with an *F*1-*score* of 0.965.

The values of the *F*2-*score* again show the same pattern as the *F*1-*score*, as both methods are derived from the precision and recall values. Since the *F*2-*score* gives more weight to recall when calculating its value, the results are slightly different. As with the *F*1-*score*, the Kernel Naive Bayes model produces the best result, with an *F*2-*score* of 0.986 at the second level of aggregation. This result correlates with the achieved recall value, since the Kernel Naive Bayes is the only model that has a recall value of 1 on the second aggregation level.

Since our main goal is to detect anomalies, it is crucial that our solution detects all attacks, even if this means accepting some false positives, as long as they are not excessive. In this context, it is important to give more weight to the recall metric than to precision. It can be concluded that Kernel Naive Bayes delivers impressive results at the second level of aggregation: 100% recall with only 101 false positives, which is well below 0.1% of all records in the dataset, and a precision of 0.933.

## 8. Discussion

### 8.1. Log Generation

Our main goal in developing a custom method for integrating different types of artifacts into logs was to efficiently create log datasets without the need for extensive computational resources, such as dedicated testbeds, and to improve the relevance of the dataset to a given organization by extending real-world logs from their environment. It is true that our method requires a lot of manual work, but, at the same time, it also requires a lot of effort in creating logs with a dedicated testbed. In comparison, the modules that are created once with our method and used to simulate different tools can be reused. With our method, any organization can seamlessly insert attack logs into their own dataset, enabling comprehensive testing of security information systems in their specific environment and facilitating the training of security personnel.

However, there are limitations and underlying assumptions to consider. First, our approach requires well-documented firewall policies. Otherwise, the log records would have to be generated directly by the firewall and not simulated by the policy documentation. Second, it assumes that the network is stable, i.e., that it is not near congestion, since adding network traffic near congestion could generate additional log artifacts. The third assumption refers to consistent network properties within the modified log sections, assuming that no significant changes have been made to the architecture or services. Finally, it is assumed that the simulated attacks do not cause significant side effects that would cause similar changes in the logs as those mentioned in assumption three. It can be argued that attacks that cause significant changes in the network have already reached an advanced stage where detection is trivial and the use of anomaly detection no longer provides any additional benefit.

An exception to the fourth assumption is the theoretical simulation of highly deterministic network-altering attacks, such as a wiper attack that renders workstations inoperable. These attacks could theoretically be mimicked by introducing certain C2 communication functions and removing subsequent log entries associated with the affected workstations, thereby mimicking their shutdown.

There is also the problem that, in the firewall logs, only the part of the communication that goes through the firewall is logged. This is the limitation of the data source that is also to be expected for other datasets where part of the communication is not visible. With this in mind, it is to be expected that the people managing the target company’s IT system will set the security controls correctly.

Experiments conducted for demonstrating the feasibility of the proposed method for generating and injecting anomalies are not sufficient to validate the quality of the logs produced. As a next step, we propose an experiment in which multiple log datasets with identical attacks are created using a subset of the methods from Figure 1, as well as the proposed method from Figure 2 [2]. If the generated datasets contain the same attacks and common anomaly detection algorithms achieve almost identical results, one could claim that the logs generated using the proposed method are of high quality and do not differ from logs generated using conventional approaches. If the quality of the generated logs can be validated, they could also be used for many other purposes, e.g., for the alert correlation approaches studied in [9]. Moreover, one of the main purposes we would like to explore in the future is a real-world scenario where the presented approach is used for training Security Operations Center (SOC) analysts, which could be done by generating and injecting logs in real-time.

### 8.2. Anomaly Detection

When comparing the unsupervised results without choosing the threshold, the average AUC PR values were the best at the second level of aggregation, and the HBOS model provided both the best average results at all levels of aggregation and the best individual result of 0.25 AUC PR at the third level of aggregation. Saito and Rehmsmeier [81] provided the baseline value for this measure, which is determined as $N_{p}/\left(N_{p}+N_{n}\right)$, where $N_{p}$ is the number of records in the positive class (anomalous records in our context) and $N_{n}$ is the number of records in the negative class (records from the existing firewall logs in our case). Using this information, as well as Table 2, the AUC PR baseline values were set to 0.0003, 0.0006, 0.0121, and 0.0056 depending on the aggregation level. Given these baseline values, almost all tested models outperformed the baseline value at all aggregation levels, indicating their superiority over a random model based on this criterion. On average, the largest difference was observed in the unaggregated data, while the smallest deviation occurred at the second aggregation level.

In experiments consisting of threshold selection with *F*1-*score* optimization, a consistent improvement in both *F*1-*score* and *F*2-*score* was observed for almost all models when a higher aggregation level was chosen. On the other hand, a higher level of aggregation resulted in a larger standard deviation for these performance measures between models. The most remarkable performance in our testing methodology was obtained by using the CBLOF model at the third aggregation level, resulting in *F*1-*score* and *F*2-*score* of approximately 0.5.

Experiments with the optimization of the precision value alongside the high recall value showed that, on average, the results with the lowest false positive rate were obtained without any aggregation. Of the models examined, the COPOD model provided the best result with a false positive rate of 3%. According to the study conducted by Axelsson [107], it is suggested that one false alarm in 100,000 events is the minimum requirement for an effective intrusion detection system, which corresponds to a percentage of false alarms of 0.001%. As observed in the experiment, none of the models even came close to this percentage. It can therefore be concluded that they are significantly ineffective in adequately implementing intrusion detection.

Based on the results obtained using the unsupervised models, it can be concluded that none of the tested unsupervised models are suitable for our task of detecting anomalies and cannot be used in the real world. For this reason, supervised learning was also used to generate test results. This has the advantage that these algorithms are usually less complex and provide better results than unsupervised algorithms, but carries the risk of overfitting to the given data. The problem of overfitting was mitigated by using 6-fold cross-validation. The results obtained were promising, especially at the second level of aggregation, where all supervised models except Naive Bayes achieved good *F*1-*score* and *F*2-*score*. The best model was Naive Bayes Kernel, with F-scores above 0.96.

The result of the Naive Bayes Kernel model can now be directly compared to the unsupervised models with optimized precision and high recall. For the unsupervised models, the best precision value was just below 0.1, while the supervised Naive Bayes Kernel model achieved a perfect recall value with a precision of 0.93. These results clearly show the superiority of the supervised models. Finally, the Naive Bayes Kernel model also fulfilled the criterion proposed by Axelsson [107] and achieved a false positive percentage of about 0.0009%.

Of all the results provided, three models stood out the most, namely CBLOF, HBOS, and Isolation Forest. This is based on the criteria that these models provided the best results, on average, across all aggregation levels in terms of AUC PR score, *F*1-*score*, and *F*2-*score*, and also performed the test methodology in reasonable time.

In contrast to the three similar comparative studies [71,72,73] identified in the literature review [8], which compared, at most, six different ML methods [73], our study examined many more ML methods, both supervised and unsupervised. In addition to the ML methods used, there are two other important aspects to consider in a comparative study: the dataset and the performance measures used in the evaluation. We believe that the dataset used for the evaluation must fulfill the following criteria in order to be used in the real world:

- The dataset used should not come from the simulated network environment, but should be generated from real network activities.
- The size of the dataset must be large enough to replicate real-world scenarios (often more than one million connections per day), as the size of the dataset strongly influences the selection of machine learning algorithms to be used.
- The proportion of anomalous records in the dataset is an important factor and should be very low (less than 1%), as anomalies in real network traffic are very rare and well hidden.

Based on these criteria, we can conclude that neither the work of Abdulhammed et al. [71] nor that of He et al. [73] fulfill the above criteria. However, He et al. [73] met all of the above criteria by using the HDFP dataset for the evaluation.

When comparing different unsupervised anomaly detection methods, it is best to use a performance measure that is independent of threshold selection. Due to the expected scarcity of anomalies compared to the prevalence of normal records in a dataset, we suggest using the AUC PR score when comparing unsupervised methods. On the other hand, when comparing supervised methods, we recommend using the *F*1-*score* or even the *F*2-*score* if you are more concerned with detecting all anomalies, rather that a lower number of false positives. We also emphasize the importance of considering the percentage of false positives relative to the total number of connections as a key indicator of the quality of the model. Of the papers analyzed, only He et al. [73] used the *F*1-*score* for supervised evaluation, while the other two papers mainly relied on the Accuracy and Detection Rate measures. Although He et al. [73] used the *F*1-*score* for the comparison, both unsupervised and supervised methods were compared, so the AUC PR score should be used for the comparison of the unsupervised methods.

## 9. Conclusions

This paper focuses on the use of a novel method to create logs containing attack-related records [2] and their use in combination with different anomaly detection methods to validate this approach [1]. The log generation method presented relies on real-world logs from the organization, augmented by domain knowledge, and eliminates the need for a dedicated testbed or installed security controls. A key advantage of this method is the ability to create logs that closely resemble the original environment. With this method, any organization can seamlessly insert attack logs into its own dataset, enabling comprehensive testing of security information systems in its specific environment and facilitating the training of security personnel.

The anomalies generated with the innovative method simulate real attacker behavior and were integrated into the existing firewall logs of a large industrial control network. To validate this approach, a comparative evaluation using different anomaly detection algorithms was performed. The integration of the injected anomalies enabled the evaluation of unsupervised anomaly detection algorithms, ensuring a comprehensive assessment of their effectiveness in detecting specific attack sequences. Furthermore, the integration of anomalies facilitated the application of supervised learning methods. As a result, both unsupervised anomaly detection models and supervised methods were thoroughly tested to assess their respective performance.

The results of this study show that none of the investigated unsupervised learning algorithms performed satisfactorily in detecting anomalies. This result confirms that the injected anomalies represent attack steps that are disguised and well integrated into the pre-existing firewall logs. However, the use of supervised learning methods provided significantly better and satisfactory results and shows the potential of the presented method to generate and integrate anomalies for anomaly detection.

With the use of the presented method, a system with a variety of potentially dangerous attack sequences can be constructed. For each of these sequences, anomalies can be generated and classifiers can be trained. Classifiers can then test the new logs received from the firewall and provide us with the probability that the specific attack sequence occurs in these logs. Based on this probability, potentially anomalous behavior can be identified and the individual steps of the attackers in the network in question can be shown.

Although the experiments demonstrated the feasibility of the proposed method for generating and injecting anomalies into logs, more research is needed to evaluate the quality of the results. For a qualitative evaluation, we proposed an experiment in which two datasets are created, one using a conventional method such as a testbed, and the other using the proposed method to inject logs. Evaluation would then be performed by comparing the results of using the two datasets for anomaly detection experiments.

## Figures and Tables

**Table 1:** Descriptive statistics of the two days of firewall logs used.

	Source IP	Source Port	Destination IP	Destination Port	Protocol
Day 1 Count	7,972,381	7,972,381	7,972,381	7,972,381	7,972,381
Day 1 Unique	1931	63,629	987	1578	3
Day 1 Top	143.198.132.18	99,999	143.239.7.57	53	tcp
Day 1 Freq	1,403,074	586,498	629,729	1,694,203	4,639,076
Day 2 Count	8,422,174	8,422,174	8,422,174	8,422,174	8,422,174
Day 2 Unique	1991	64,167	1308	1606	3
Day 2 Top	143.198.132.18	99,999	143.239.7.58	53	tcp
Day 2 Freq	1,660,695	579,462	758,855	1,756,672	5,035,476

**Table 2:** Statistics of the number of normal, anomalous, and total records in each dataset used in the unsupervised test method for each aggregation level.

	Day 1			Day 2		
**Aggregation Level**	**Normal**	**Anomalous**	**Total**	**Normal**	**Anomalous**	**Total**
Unaggregated	7,972,381	2327	7,974,708	8,422,174	2327	8,424,501
First level	3,141,435	2054	3,143,489	3,372,934	2054	3,374,988
Second level	114,104	1411	115,515	116,395	1411	117,806
Third level	90,091	513	90,604	91,617	513	92,130

**Table 3:** Comparison of performance measures for the test set using the best six supervised learning models at the second and third levels of aggregation.

	Second Level of Aggregation				Third Level of Aggregation			
**Model Name**	**Recall**	**Precision**	**F1-Score**	**F2-Score**	**Recall**	**Precision**	**F1-Score**	**F2-Score**
Naive Bayes	0.9986	0.0052	0.0104	0.0256	1	0.0206	0.0403	0.0950
Kernel Naive Bayes	1	0.9331	0.9654	0.9859	1	0.6905	0.8169	0.9177
Logistic Regression	0.9008	0.8826	0.8916	0.8971	0.61	0.9973	0.757	0.6614
Random Forest	0.8689	0.9983	0.9291	0.8920	0.6413	1.0000	0.7815	0.6909
Decision Tree	0.8696	0.9943	0.9278	0.8920	0.1423	0.9985	0.2491	0.1718
Gradient Boosted Trees	0.8696	0.9895	0.9257	0.8912	0.6608	1.0000	0.7958	0.7089

## Data Availability

Data are contained within the article.
